# Metabolic shifts: a fitness perspective for microbial cell factories

**DOI:** 10.1007/s10529-012-1038-9

**Published:** 2012-08-31

**Authors:** Anisha Goel, Meike Tessa Wortel, Douwe Molenaar, Bas Teusink

**Affiliations:** 1 Systems Bioinformatics IBIVU, Vrije Universiteit Amsterdam, De Boelelaan 1085, 1081 HV Amsterdam, The Netherlands; 2 Laboratory of Microbiology, Wageningen University and Research Centre, Dreijenplein 10, 6703 HB Wageningen, The Netherlands; 3 Kluyver Centre for Genomics of Industrial Fermentation/NCSB, P.O. Box 5057, 2600 GA Delft, The Netherlands

**Keywords:** Biotechnology industry, Evolution, Fitness, Metabolic shift, Systems biology, Trade-off

## Abstract

Performance of industrial microorganisms as cell factories is limited by the capacity to channel nutrients to desired products, of which optimal production usually requires careful manipulation of process conditions, or strain improvement. The focus in process improvement is often on understanding and manipulating the regulation of metabolism. Nonetheless, one encounters situations where organisms are remarkably resilient to further optimization or their properties become unstable. Therefore it is important to understand the origin of these apparent limitations to find whether and how they can be improved. We argue that by considering fitness effects of regulation, a more generic explanation for certain behaviour can be obtained. In this view, apparent process limitations arise from trade-offs that cells faced as they evolved to improve fitness. A deeper understanding of such trade-offs using a systems biology approach can ultimately enhance performance of cell factories.

## Introduction

Among the several microorganisms used in the food and biotechnology industry, *Escherichia coli*, by far the most widely studied microorganism, is an excellent work-horse for the production of several high value products (Table 1). Other work-horses include *Bacillus subtilis*, lactic acid bacteria, yeast (*Saccharomyces cerevisiae*), fungi (*Aspergilli*) and mammalian cell lines, each utilized for the production of a wide range of products that are directly or indirectly an inherent part of our daily lives.

**Table 1:** Summary of various organisms used as industrial work horses, the shifts in metabolic strategies they exhibit, their industrial applications and the mechanisms of regulation

Microorganism	Metabolic shifts/trade-offs	Application	Mechanism of regulation
*Escherichia coli*		Recombinant proteins (Leuchtenberger et al. 2005), amino acids (Park and Lee 2010), vaccines (Shiloach and Rinas 2009) and immobilized enzymes (Synowiecki et al. 2006)	Limitations in the carboxylic acid cycle due to limited oxygen and carbon source availability, tight regulation of the CoA pool and environmental conditions (Wolfe 2005) Redox ratio: need to regenerate NAD^+^ in the absence of oxygen (Vemuri et al. 2006) Global regulators (CcpA, CodY and TnrA) exerting control at the transcriptional level of catabolic genes and operons (Fujita 2009; Sonenshein 2007; Stülke and Hillen 2000) Phosphoenolpyruvate-pyruvate-oxaloacetate node dynamics (Sauer and Eikmanns 2005)
*Bacillus subtilis*		Vitamins, heterologous proteins and enzymes (Pohl and Harwood 2010; Shimizu 2008)	
Lactic acid bacteria		Dairy and fermented foods, probiotics, bulk and fine chemicals (Teusink and Smid 2006)	Triggered by carbon source limitation (Thomas et al. 1979) and oxygen concentration (Jensen et al. 2001) Balance of the NADH/NAD^+^ ratio (Cocaign-Bousquet et al. 1996) Allosteric effects of fructose-1,6-bisphosphate (FBP) and triose phosphates on mixed acid branch enzyme activities, inhibition of alcohol dehydrogenase by adenine nucleotide pool (Neves et al. 2005) Modulations of certain transcripts and protein levels (Kowalczyk and Bardowski 2007)
Yeast (*Saccharo*-*myces cerevisiae*)		Baking, brewing, wine-making, bioethanol, bulk and fine chemicals, recombinant proteins (van Dam et al. 2002; Nevoigt 2008)	Low affinity and high capacity of pyruvate decarboxylase compared with pyruvate dehydrogenase enzymes (Postma et al. 1989; Pronk et al. 1996) Post-translational regulation (Daran-Lapujade et al. 2007; Pronk et al. 1996) Differential gene expression (Pronk et al. 1996) Flux-sensing via FBP (Huberts et al. 2012) Balance of the NADH/NAD^+^ ratio (Vemuri et al. 2007)
Filamentous fungi (*Aspergilli*)		Proteins, enzymes bulk and fine chemicals (Meyer et al. 2011)	Environmental influences triggering transcriptional regulation Regulation by global regulators Sporulation associated signal transduction (Hoffmeister and Keller 2007)
Mammalian cell lines (Myeloma, Hybridoma, etc.)		Recombinant proteins, monoclonal antibodies, nucleic acid-based drugs (Lim et al. 2010; Reiter and Blüml 1994; Vives et al. 2003)	Warburg effect: lactate production via enhanced glycolysis despite the presence of adequate oxygen (Warburg 1956) Increase in glucose transporters and kinases, post-translational modifications of enzymes, hypoxia-inducible factor: HIF, mitochondrial defects (Gatenby et al. 2010; Gatenby and Gillies 2004; Gillies et al. 2008; Gillies and Gatenby 2007) Regulation by metabolic enzymes (Diaz-Ruiz et al. 2011)

Not all of these organisms had the complete set of desired traits to start with. Multiple methods are employed to obtain the preferred properties, including evolutionary engineering, classical mutagenesis and screening, rational and reverse metabolic engineering, global transcription machinery engineering or genetic modification (Nevoigt 2008), and more recently synthetic biology (Khalil and Collins 2010). Numerous successes in substantial improvement of processes and strains have been reported in the past decades (Brockmeier et al. 2006; Donalies et al. 2008; Ikeda 2006; Park and Lee 2010; Smid et al. 2005). Nevertheless, common practical problems are encountered due to the shifts in metabolic strategies during growth (Table 1).

Industrial strains need to have and retain the required properties to maintain high production rates. However, the one process that none of these strains can evade is their evolution, governed by their “fitness” in the respective environments. Microorganisms are subject to selection and the selection pressure is often on specific growth rate. In a fermentor the fastest growing strain produces the most progeny and therefore is likely to invade most of the population. How well microorganisms flourish in terms of competing with other strains, is called their *fitness*. Most often, the strain properties necessary for industrial production processes are not the same as those that enable the cell to attain maximal fitness. Hence, identifying the selection pressures and strategic decisions that microorganisms can make, will help in tuning their environment so as to align their cellular objectives with the production process objective, and ensure constancy in biotechnological applications.

## Understanding physiology from the perspective of optimized fitness

The end result of microbial physiology is a direct consequence of adaptations that improve fitness, which can be mimicked in silico by adopting some optimality criterion for a microorganism in its environment. The premise of this approach is that cells will adapt, often surprisingly fast, and move towards some optimal fitness if cultivated under constant conditions. Such an in silico optimality approach has been used frequently over the years, and is often also disputed: microorganisms might not be optimal for specific tasks. At the end of this section, we will show a counterexample of this optimality assumption.

Nearly two decades ago, physiological observations of *E. coli* were explained by optimization of growth within stoichiometric constraints (Varma et al. 1993) using the well-known modelling approach for analysing biochemical networks: Flux Balance Analysis (FBA) (Orth et al. 2010). In the post-genome era, this approach was extended to genome-scale metabolic networks. An early example successfully demonstrated that optimizing metabolic network fluxes to maximize growth could explain physiological metabolic behaviour in *E. coli* (Edwards et al. 2001). In this approach, measured nutrient uptake rates are used to constrain the metabolic network which is then optimized for maximal growth, to generate predictions of growth and product formation rates. The in silico predictions of growth of *E. coli* on acetate and succinate were found to be consistent with experimental measurements. Microorganisms are thus limited by environmental constraints and the aforementioned studies reinstate that the resulting physiological behaviour is a consequence of an underlying optimality objective which improves their fitness.

**Fig. 1:**
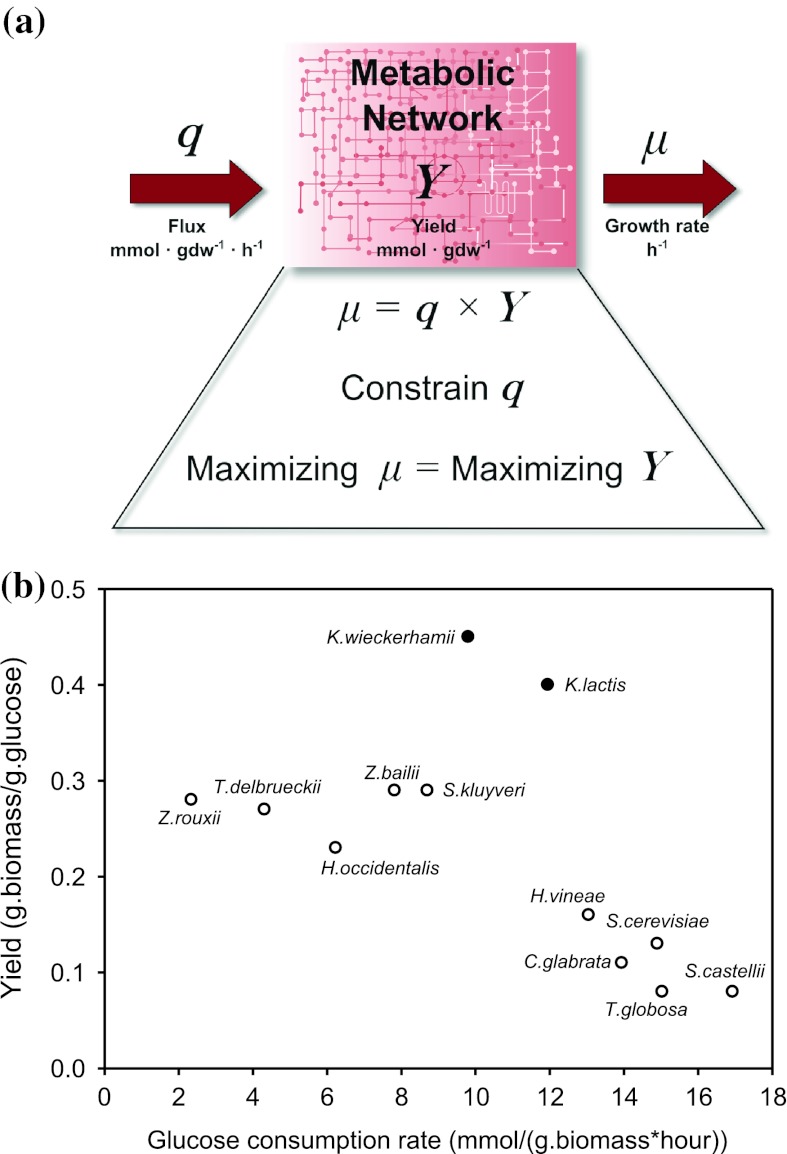
Yield and rate. **a** Why flux balance analysis (FBA) is in fact a yield optimization problem rather than a rate optimization problem. **b** Trade-off between biomass yield and substrate uptake rate for a number of exponentially growing yeast species: Reprinted by permission from Macmillan Publishers Ltd: [Heredity] (MacLean 2008)

However, not all physiological states can be described by growth optimization. This is because under varying environmental settings, cells often exhibit suboptimal behaviour where their resulting growth rate is very different from what a standard FBA would predict. Schuetz et al. (2012) showed that a multidimensional objective can attempt to explain suboptimal behaviour. Additionally, as pointed out by Teusink et al. (2006), growth optimization in FBA is in fact yield optimization (Fig. 1a) and therefore in scenarios where yield optimization is not the objective, standard FBA approaches will invariably fail to predict observations (Santos et al. 2011; Schuster et al. 2008). This is to be expected for biotechnologically relevant conditions such as high concentrations of rapidly fermentable sugars that lead to ATP-inefficient metabolism. Indeed, in the seminal paper from the group of Palsson, it was shown that *E. coli* evolves towards an in silico predicted “line of optimality” on glycerol, but, on glucose, the evolved cells increased their growth rate but moved away from the FBA-predicted line of optimality by producing acetate (Ibarra et al. 2002). The same difference between glucose and glycerol was observed for *Lactobacillus plantarum* (Teusink et al. 2006, 2009).

FBA applies only a limited set of constraints, being mass-balance constraints (steady state assumption) and some capacity constraints (usually on input fluxes) to bound fluxes through the network. New approaches which apply additional constraints routed in physics and chemistry have to be used to understand metabolic strategies that FBA cannot explain. Beg et al. (2007) for the first time, used the macromolecular crowding or solvent capacity constraint on the metabolic network of *E. coli*. This constraint limits the total intracellular space available for enzymes in cytoplasm. With this constraint, FBA was able to reproduce acetate production in *E. coli*. Subsequently, this approach was used to model proliferating mammalian cells to explain the Warburg effect (Shlomi et al. 2011; Vazquez et al. 2010). These approaches extend the notion of metabolic efficiency being analogous to stoichiometric ATP-yield only: different flux distributions have different implementation consequences (costs if you will), that should also be taken into account when computing optimal behaviour, as we will elaborate on later.

### Flux balance analysis (FBA) of multiple species

Microorganisms seldom live in isolation and analysing single species metabolic networks in isolation provides little insight into microbial interactions in communities. Consequently there have been recent efforts to model competition, co-existence, and strain and species interactions using multispecies stoichiometric metabolic modelling. Zomorrodi and Maranas (2012) recently developed a comprehensive FBA framework, OptCom, capable of capturing the trade-offs between individual and community fitness criteria. This approach uses a multi-level, multi-objective optimization routine that allows for constraints of individual species in a larger scaffold of community-level objective maximization. The authors use genome-scale metabolic models of a two-species microbial system and quantify the syntrophic interaction in terms of the extent and direction of transfer of metabolites and electrons between species. Simpler approaches were also used to predict metabolic fluxes, interspecies electron transfer and the ratio of constituent species for anaerobic microorganisms (Stolyar et al. 2007) and in subsurface environments (Zhuang et al. 2011a). Tzamali et al. (2011) used a graph-theoretic approach to identify metabolic interactions and their importance on growth in *E. coli* strain communities. Their results suggest that in certain communities, cross-feeding enhances the growth rate of participating species. The main issues in all of these approaches, that are currently being actively investigated, are how to balance fluxes that are catalyzed by species with different abundances in the population, and what would be a realistic objective for such a community. In summary, multispecies metabolic modelling is an emerging field that aims to quantify metabolic interactions, identify trade-offs and to provide insights into the impact of different substrate availability on species abundance in microbial communities. Some powerful approaches are starting to develop and are getting ready for use in biotechnological applications.

### Cheaters and unexpected strategies in communities

At times, the outcome of optimization of microbial fitness can be surprisingly intricate: an important additional attribute of the optimum is that it should be (evolutionarily) stable. In one such example, *Lactococcus lactis* excretes an extracellular protease to degrade milk proteins into free utilizable peptides, a feat required when the peptides in the environment are insufficient for growth. Under these conditions, one would intuitively expect this trait to be selected for. However, this protease is extracellular and the peptides produced do not merely benefit the cell secreting the protease, but in part also diffuse away from it, becoming accessible to neighbouring cells. To grow well, it would indeed be beneficial if all cells produce this protease, but imagine a scenario where one cell does not. This “cheater” cell will still consume peptides released by neighbouring cells but will have more resources (not allocated to protease production) available for growth and reproduction. This, on average, will lead to more progeny and a spread of the protease-negative trait in the population. In fact, it was shown experimentally that this leads to a population that completely loses the protease-positive trait and depending on the conditions, grows much slower (Bachmann et al. 2011). A similar study in yeast showed that the trait for enzymatic breakdown of sucrose by secreted invertase is selected against, because the glucose and fructose formed thereafter diffuse away, and can be used by other individuals (Gore et al. 2009). This is a very counter-intuitive outcome of the effect of selection on the physiology of a species, even under constant conditions. A detailed theoretical analysis of this cooperative and cheating behaviour and its implications on biotechnological applications was reviewed recently (Schuster et al. 2010).

## Trade-offs: the role of physical and biochemical constraints

In the previous section we discussed a modelling framework (FBA) using empirically derived uptake flux constraints and additionally an intracellular space constraint. The latter results in a shift, from efficient use of potential chemical energy in the substrate through oxidative phosphorylation to inefficient use through aerobic glycolysis, in a model of human cancer cells (Shlomi et al. 2011; Vazquez et al. 2010). In this example there are *constraints* (to obtain a certain flux some intracellular space is required) and *limits* (there is a limited amount of intracellular space), which necessitate a choice between oxidative phosphorylation and aerobic glycolysis and this we call a *trade*-*off*. Essentially, trade-offs call for a choice between two incompatible features, either of which if chosen, automatically leads to forfeiting the other. There are several biological examples of trade-offs: cells can invest in growing bigger or producing new cells, cells can be optimized for their current environment, or be prepared for possible future changes, just to mention a few.

There could be similar constraints and limits that influence the uptake rate. For example, retaining membrane integrity requires a certain percentage of lipids (Molenaar et al. 2009) and there might be restrictions on the kinetic constants of enzymes (Heinrich et al. 1991). A limit on the uptake flux might arise because higher uptake flux requires more transporter synthesis that is limited by availability of precursors and cellular machinery. Hence, to answer ‘why’ organisms regulate their metabolism, one needs to identify constraints that actually limit cellular function, namely, physical or biochemical constraints. These constraints can stem from thermodynamic laws, solubility of proteins or stability of DNA. More information about these constraints is rapidly becoming available on web-databases like BioNumbers (Milo et al. 2010). Furthermore, these constraints that govern trade-offs also have an origin in the physics of biological materials. As we try to find these more profound explanations, rather than taking observed constraints for granted, we also obtain a more fundamental understanding of observed cellular behaviour.

## Trade-offs in microbial and industrial processes

Some trade-offs are relatively obvious, such as the examples discussed in the previous section. Occasionally, however, a trade-off appears indirectly because we observe species specialized in one trait or in another trait, but never in both. One less obvious trade-off is the one between catabolic rate and ATP-yield (Pfeiffer et al. 2001). This trade-off is well described for a metabolic pathway (Aledo and del Valle 2002; Angulo-Brown et al. 1995; Waddell et al. 1997). In a pathway, the free-energy of the substrate can be used either to produce high free-energy intermediates or to drive the pathway quickly, making yield and rate incompatible features. But does this argument also hold for the trade-off between catabolic rate and ATP-yield, considering the numerous pathways and cellular processes involved?

Several microorganisms exhibit inefficient (low-yield) metabolism during fast growth. Above a critical growth rate and corresponding glucose concentration, *S. cerevisiae* ferments glucose (Postma et al. 1989). A similar metabolic shift to a regime with decreasing ATP-yield and increasing catabolic rate is observed in lactic acid bacteria (Thomas et al. 1979) and in mammalian cells (see table 1). MacLean (2008) showed that biomass yield plotted against glucose consumption rate of several exponentially growing yeast species shows a negative slope, with none present at the high yield high consumption region (Fig. 1b), suggesting a trade-off between catabolic rate and ATP-yield.

Trade-offs in industrial processes are not uncommon either, the most classic one being the choice between batch and continuous fermentation. Batch fermentations bear a lower contamination risk and a higher cost due to additional cleaning cycles, whereas continuous fermentations offer the advantages of steady-state operation, longer runs with shorter downtimes, better product consistency, easier process control, and steady utility demands (Shuler and Kargi 2002; Wang et al. 2005). But because continuous fermentations run longer, and cells might experience selection pressures different from those previously experienced, the cells will evolve. This can lead to undesirable side-effects and loss of strain productivity (Douma 2010). Another trade-off is seen in the dairy industry, where yogurt production requires strains that excrete exo-polysaccharide (EPS) for good texture and mouth-feel. But this trait leads to higher viscosity that can be quite problematic during starter culture production due to difficulties in downstream processing. Hence a single application entails two conflicting objectives. A similar trade-off exists for the production of cheese-starter culture and yeast. The final use of these cultures is the production of lactic acid and flavour compounds for cheese, ethanol for beverages, and CO_2_ for fluffy breads. However, during the initial start-up or growth phase of the fermentation process as well as for starter culture suppliers, the aim is to maximize biomass production without compromising adequate functionality of the resulting strain. Thus growing fast with high biomass yields versus achieving high levels of end products represents a trade-off.

To predict the outcome of evolution, merely identifying a trade-off is insufficient, since we still do not know which incompatible trait the strain will specialize in. For instance, at high substrate concentrations, species will evolve towards higher growth rates, and—one may suspect—a low biomass yield. Alternatively, when the selection pressure is for a high yield, as is the case for cells in biofilms living in close proximity with their relatives (Kreft 2004), species will attain a high yield but probably a lower growth rate. That the evolution of species depends on the selection pressure exerted by the environment is important to realise when evolving species in the laboratory or improving strains for bio-industry, because an environment that improves one trait might compromise another. Thus, to improve a trait, it becomes extremely important to find conditions with the *right* selection pressure. A fascinating example illustrating this is improving accumulation of storage polymers via feast-famine cycles (Chiesa et al. 1985; van Loosdrecht et al. 1997). The condition comprises subjecting cells to cycles of short-time in high substrate environment and long-time without substrate. This condition selects for cells that store substrate during the feast regime and use it in the famine regime.

## From regulatory mechanisms to the underlying generic causation: fitness

A plethora of regulatory mechanisms involved in causing and regulating metabolic shifts in various organisms exist in the literature (see Table 1 for a brief summary). These studies have provided a wealth of knowledge in understanding metabolic shifts. While it is crucial to identify the regulatory and molecular mechanisms of metabolic shifts, they are different instantiations of the same phenotype that these cells seems to be selected for. In cancer this is most obvious: whilst different tumours have vastly different mutations, most of them display the Warburg effect (Hanahan and Weinberg 2011). Therefore, besides identifying the mechanisms of metabolic shifts, we want to find a global explanation of why we see certain patterns of behaviour. In order to get a better understanding of its long-term behaviour it is also important to think about ‘why’ such a regulation system arose in the first place, in other words, what contribution it had to the fitness of the organism.

**Fig. 2:**
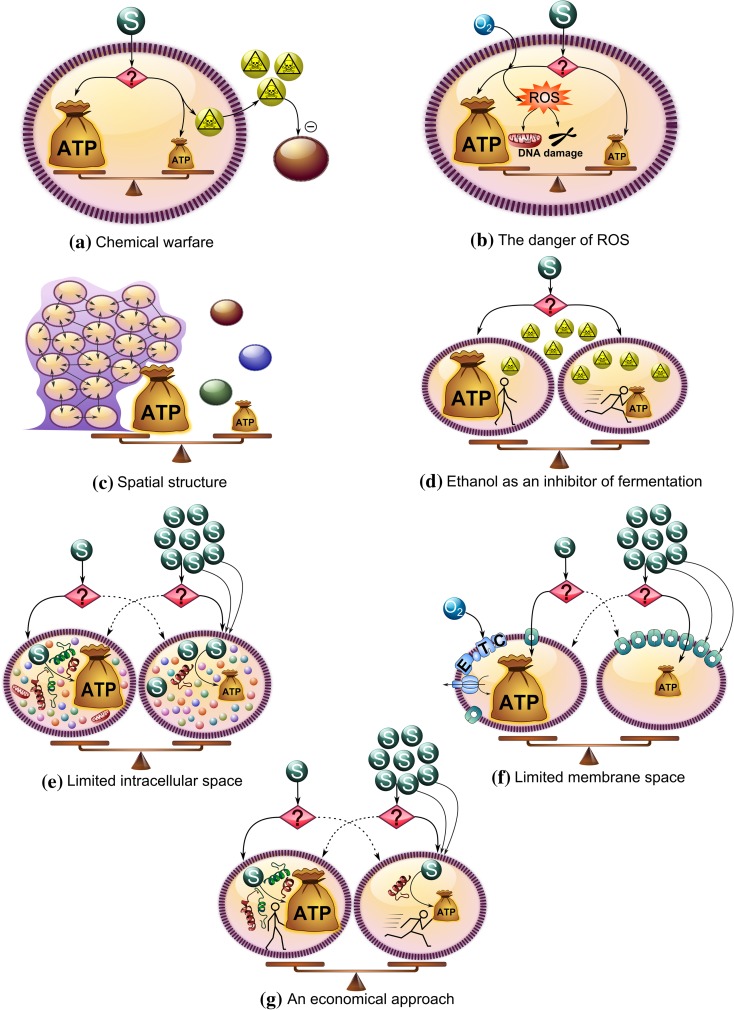
Different hypotheses and trade-offs involved, for growth rate (and substrate (S)) related ATP-efficient and inefficient metabolism. **a** Chemical warfare: at the cost of ATP production, toxic compounds are produced in order to inhibit the growth of competitors. **b** The danger of reactive oxygen species (ROS): additional ATP production via respiration concomitantly generates ROS that can damage DNA. **c** Spatial structure: spatial structure promotes ATP-efficient substrate usage but lone individual cells can grow faster as long as sufficient substrate is available. **d** Ethanol as an inhibitor of fermentation: substrate can be used efficiently but slowly or fast but inefficiently and the latter strategy produces toxic compounds that are exported but nonetheless accumulate more inside the cells producing them. **e** Limited intracellular space: due to limited intracellular space and bulky respiratory machinery, the flux through respiration cannot match high substrate uptake rates and a gradual shift to inefficient metabolism occurs. **f** Limited membrane space: the membrane can be used to produce additional ATP from substrate via the electron transport chain (ETC.) or to take up more substrate. **g** An economical approach: substrate can be used slowly and efficiently but this requires a lot of proteins, or it can be consumed fast but inefficiently which requires much less proteins

As we saw earlier, trade-offs might be an underlying cause for metabolic shifts, but identifying the key trade-offs can be difficult. Several explanations suggested for growth-rate-related metabolic shifts in microorganisms are discussed in subsequent sub-sections (Fig. 2). The advantage of the ATP-efficient pathway seems relatively clear because it produces more energy per substrate. We will therefore first discuss explanations for the use of ATP-inefficient pathways.

### Chemical warfare

End products of inefficient metabolism are often toxic and inhibit growth of neighbouring species, for instance, in lactic acid bacteria (Loesche 1986) and yeast (Piskur et al. 2006). Groups of microorganisms, at a cost of reduced efficiency, produce these inhibitory compounds to reduce competition (Fig. 2a). However, if a mutated cell uses the ATP-efficient pathway in an inefficient population, it could gain higher fitness. This is because its neighbours would still produce ethanol and intoxicate competitors, and the efficient mutant would benefit from the toxic effect on the population without itself bearing the burden of producing ethanol, thereby gaining an advantage with higher ATP availability. But this ATP-efficient strain should—under the assumption in this scenario—grow faster and take over the population, a fundamental flaw in the hypothesis of “chemical warfare”.

Yeast can also use its fermentation product, ethanol, as a substrate. Based on this observation, a make-accumulate-consume strategy comprising first producing ethanol and later consuming it when glucose is depleted was proposed (Piskur et al. 2006). Such behaviour is also seen in *E. coli* (Koser 1923) and *B. subtilis* (Speck and Freese 1973) and suggested in lactic acid bacteria that can use mixed acid fermentation products as substrate (Hols et al. 1999). This strategy may seem clever but, if the cells waste part of the energy obtainable from the substrate to accumulate fermentation products for later consumption, they will have a lower fitness if they never encounter glucose depletion. In addition, there could be “cheaters” not producing, but consuming ethanol produced by others. This hypothesis also seems to suffer from the same cheater-invasion problem as the chemical warfare hypothesis does.

### The danger of reactive oxygen species

At high growth rates, though respiration is more ATP-efficient, it could also have serious disadvantages leading to prohibitive constraints. A putative issue with respiration is the formation of reactive oxygen species as a natural by-product (Fig. 2b). In yeast and mammalian cells, cells can ferment during the DNA replication phase because respiration causes DNA damage (Anastasiou et al. 2011; Chen et al. 2007). This does not directly explain why cells respire during slow growth, although time spent on DNA replication is much less at lower growth rates. But it is a challenge to determine whether increase in DNA replication time and metabolism at high growth rates quantitatively explain shifting to fermentation, because the dependency of ROS production on respiration is rather complicated (Kowaltowski et al. 2009).

The previous hypotheses address the prevalence of inefficient metabolism due to the useful impact of its by-product(s) or the negative impact of efficient metabolism. The following explanations all assume a trade-off between growth yield and growth rate. Subsequently, if the selection pressure acts on growth rate, only inefficient pathway usage is expected to prevail, simply because it is faster. Under such presumptions, the use of *efficient* metabolism at low growth rates needs to be explained!

### Spatial structure

Modelling efforts show that the existence of spatial structure in a population (due to incomplete mixing or biofilm formation) can select for efficient metabolism (Aledo et al. 2007; Kreft 2004; Pfeiffer et al. 2001) because it increases substrate availability, benefiting closely-related neighbours (Fig. 2c). Inside these non-motile populations, cheater cells using inefficient metabolism might still evolve, but if cells disperse often enough to start a new colony, efficient metabolism can still prevail (Kreft 2004). Cooperation with related cells is even stronger for multicellular organisms, except obviously for cancer cells. Experiments confirm that spatial organization promotes efficient metabolism while well-mixed cultures sustain inefficient metabolism (MacLean and Gudelj 2006). Nonetheless, even in well mixed cultures, efficient to inefficient metabolism shift is observed (Hollywood and Doelle 1976; Postma et al. 1989; Snay et al. 1989; Thomas et al. 1979), rendering this hypothesis incomplete, if not questionable.

### Ethanol as an inhibitor of fermentation

In a competition experiment between fermenting and respiring yeast cells, addition of extracellular fermentation products had a negative influence on the fermenters (MacLean and Gudelj 2006). The presumption is that at higher extracellular ethanol concentrations, ethanol export is more difficult for fermenters, resulting in high and toxic intracellular ethanol concentrations (Fig. 2d). But higher accumulation of intracellular ethanol in fermenters in comparison with respirers is not proven yet, leaving this hypothesis open. Besides, it is unlikely that this is a universal explanation, because bacteria shifting between mixed acid and homolactic fermentation need to export either acetate and formate, or lactate, and it is unclear which products are more harmful.

So far we have summarized explanations for the use of inefficient pathways: chemical warfare and the danger of reactive oxygen species, and efficient pathways: spatial structure and toxic effects of ethanol. But often, efficient metabolism is observed at low growth rates and inefficient metabolism at high growth rates. In the forthcoming sub-sections we will review approaches that attempt to explain the metabolic shift as a function of growth rate.

### Limited space

#### Intracellular space

As described in Sect. 3, intracellular space constraints can impose a metabolic shift with increasing nutrient uptake in cancer cell models. The hypothesis is that respiration machinery requires more space and cannot match a high uptake flux, resulting in a shift to lactate production (Fig. 2e). It remains to be shown that intracellular space is indeed limiting, as cells can change size or shape to tweak the uptake relative to intracellular space.

#### Membrane space

Under varying circumstances, the electron transport chain and glucose transporters compete for the limited membrane space (Fig. 2f). Thus transport rate depends on the space occupied by transporters and the electron transfer chain in the membrane. Flux balance analysis on the *E. coli* metabolic network with this dynamic constraint predicts that maximum growth is possible with efficient metabolism at low growth rates and inefficient metabolism at high growth rates, which is in agreement with experimental results (Zhuang et al. 2011b). Thus membrane constraints can explain metabolic shifts, but only in bacteria containing efficient pathway components in their membrane, and it can perhaps be adjusted to explain the shift in eukaryotes containing limited mitochondrial membrane space. This hypothesis cannot, however, explain the shift in lactic acid bacteria involving only cytosolic enzymes.

### An economical approach

Molenaar et al. (2009) hypothesized that the metabolic shift is in fact due to a resource allocation problem for optimal fitness, with growth rate as a proxy for fitness. They introduced a self-replicator model; a simple representation of a cell with efficient and inefficient metabolic pathways that gives insight into which strategy leads to fastest growth. By taking into account that the efficient pathway actually needs more cellular machinery to operate (a longer pathway in lactic acid bacteria, an electron transport chain in *E. coli* and mitochondria in yeast), the self-replicator model predicts that at low substrate concentrations efficient metabolism leads to a higher growth rate, and at high substrate concentrations inefficient metabolism leads to a higher growth rate (Fig. 2g). This approach takes both the benefits (ATP efficiency) and the associated costs into account when considering alternative metabolic strategies and thus introduces a hypothesis for the metabolic shift as a function of nutrient availability and hence, growth rate. However, it remains to be shown that the difference in pathway costs can indeed cause this shift in optimal strategy in biological systems.

## The cycle of systems biology

**Fig. 3:**
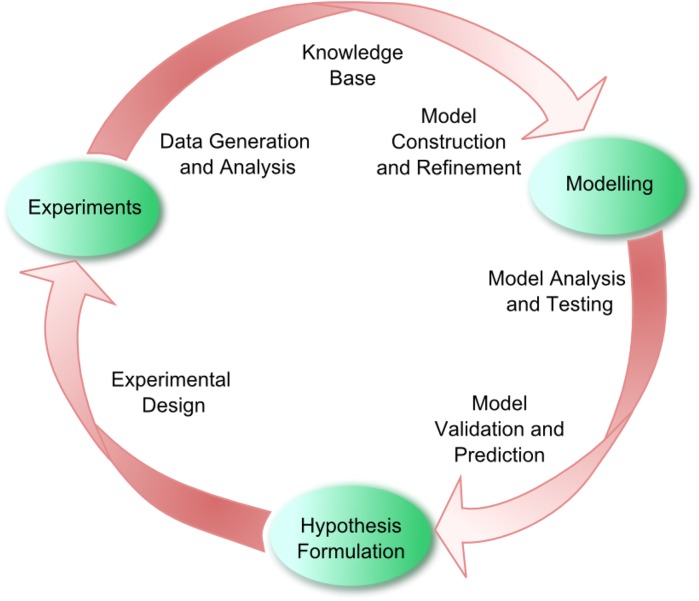
The cycle of systems biology. Defined as the quantitative study of biological processes as whole systems, instead of isolated parts, systems biology comprises utilizing knowledge bases and experimental data to develop and construct computational models to propose new hypotheses. The field is characterized by synergistic integration of data and theory that can be combined to produce a model. Model analysis leads to predictions of physiological functions which might be difficult to obtain otherwise. Validation of these predictions helps identify novel components or interactions, which in turn refine the model. Ultimately, the effectiveness of a model does not necessarily depend on goodness-of-fit, but on its usefulness in, for example, (i) providing new hypotheses/leads as predictions, (ii) providing a data integration platform as a formal representation of current knowledge, or (iii) helping to discriminate between alternative explanations

It remains a challenge to validate or falsify the hypotheses described in the previous section. Many of them look at only a specific aspect of metabolism. Nevertheless, these hypotheses call for an integrative approach, since fitness-associated costs are a systems property and cannot be inferred by studying a single component in isolation. Even then, efforts to approximate the costs of protein synthesis (Dekel and Alon 2005; Shachrai et al. 2010; Stoebel et al. 2008) have remained inconclusive. Yet, to understand microbial physiology we believe that a systems biology approach is the best, perhaps the only, option available. Systems biology aspires to capture how systems properties emerge from orchestrated interactions between individual components in an organism, using iterative cycles of quantitative experimental data generation and mathematical modelling (Fig. 3). Systems biology studies have shown the ability to address similar problems in the past. Wessely et al. (2011) incorporated genome-wide ‘omics’ data into the genome-scale metabolic network of *E. coli* using various network and optimization tools to link protein investment and transcriptional regulation of pathways. With this integrative approach they identified and suggested an evolutionary trade-off between protein investment and rapid response time. From the industrial perspective, there have been quite a number of successes in systems metabolic engineering combining systems biology, synthetic biology and evolutionary engineering principles (Lee et al. 2011). Accumulated knowledge has been used to perform guided evolution comprising a combination of clever knockouts and selection pressures to produce industrially important compounds via stable processes.

Finally and ultimately, a systems biology approach should connect environmental conditions to genes, transcriptional regulation, transcription factor interactions and protein production to metabolism in a single model. One such example exists that proposes cell regulation via flux sensing metabolites in *E. coli* (Kotte et al. 2010). This is a good example of how integrated models could look, as closed-loop systems comprising all levels in the cell. Such studies are currently restricted to model organisms such as *E. coli* as it has been studied for decades and can boast of a rich source of detailed knowledge, unlike other microorganisms. This necessitates multi-level omics studies in the latter to be able to investigate them with realistic models. There is hope that we can translate such kinetic models developed for model organisms to less-well studied organisms through what we have called comparative systems biology (Levering et al. 2012).

## Concluding remarks

We have discussed industrially-relevant examples of metabolic shifts exhibited by organisms, summarized the underlying regulatory mechanisms, emphasized the existence and role of trade-offs in these metabolic choices, and scrutinized various hypotheses and their pitfalls in explaining the fitness advantage of metabolic shifts. Systems biology, we believe, is the best approach we currently have to tackle such complexities of cell factories. Nevertheless, one must proceed with caution in the midst of current high-throughput data generation methods and avert sinking in oceans of data by regularly stepping back to recapitulate the greater objective. We firmly believe that the functional perspective, i.e. the contribution of the observed adaptive mechanisms to fitness, in the light of constraints and trade-offs, provides a powerful context to our understanding of the physiology of microbial cell factories. We are still quite at the tip of the iceberg but with constant consolidated systems biological efforts we can aim to reach a deeper understanding that will guide future major innovations in biotechnology and medicine.
